# Effect of end group of amorphous perfluoro-polymer electrets on electron trapping

**DOI:** 10.1080/14686996.2018.1477395

**Published:** 2018-06-12

**Authors:** Seonwoo Kim, Kuniko Suzuki, Ai Sugie, Hiroyuki Yoshida, Masafumi Yoshida, Yuji Suzuki

**Affiliations:** a Department of Mechanical Engineering, The University of Tokyo, Tokyo, Japan; b Graduate School of Engineering, Chiba University, Chiba, Japan; c Molecular Chirality Research Center, Chiba University, Chiba, Japan; d Faculty of Liberal Arts and Sciences, Tokyo City University, Tokyo, Japan

**Keywords:** Electret, fluorinated polymer, CYTOP, density functional theory, low-energy inverse photoelectron spectroscopy (LEIPS), electron affinity (EA), 20 Organic and soft materials (colloids, liquid crystals, gel, polymers), 104 Carbon and related materials, 202 Dielectrics / Piezoelectrics / Insulators, 401 1st principle calculations, 502 Electron spectroscopy

## Abstract

Charge trap in amorphous perfluoro-polymer electret is studied, focusing on electron trap site and trap energy. Low-energy inverse photoelectron spectroscopy is adopted to measure solid-state electron affinity (EA) of cyclic transparent optical polymer (CYTOP). EA of CYTOP CTL-S is discovered by compensating the unwanted charge-up effect. Negatively-charged electret materials (polyethylene, ethylene-tetra-fluoro-ethylene, poly-tetra-fluoro-ethylene, and CYTOP) are analyzed by quantum mechanical calculation. Density functional theory with long-range correction is adopted to analyze orbital energies of single molecular systems. Intramolecular distribution of trapped electron and EA are investigated. Calculated electron affinities of CYTOP polymers with different end group are qualitatively in accordance with trapped charge stability measured with thermal stimulated discharge, signifying that electron affinities obtained with the present simulation can be used as an index of amorphous polymer electret.

## Graphical abstract

**Figure:**
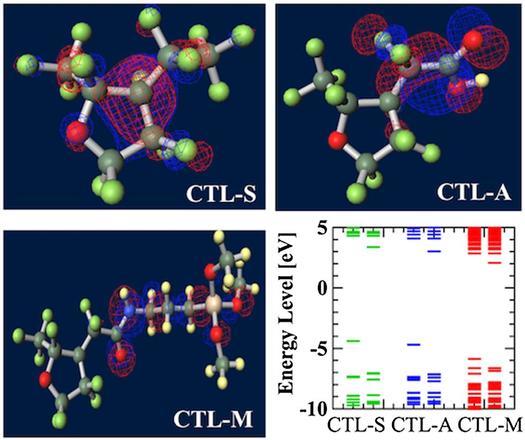

## 1. Introduction

Electret is dielectric with quasi-permanent charges. The charges trapped in proper electret materials are able to generate electrostatic field for tens of years [1]. Various electro-acoustic/electro-mechanical electret transducers have been proposed and realized [1,2]. Power generation using electrets was proposed decades ago [3], and in recent years, electret-based energy harvesting has come to the fore as a long-lasting power source for low-power electronics [4]. In vibration-driven electret energy harvesters, electret is prepared on an electrode and charges with the opposite sign are induced on the counter electrode. From relative motion between electrodes by external vibration, the capacitance as well as the amount of induced charges is changed, leading to induced current in the external circuit. Development of the high performance electret has been desired, since the power output of the harvester is proportional to square of the surface potential [4]. Electret materials are grouped into polymer electrets and inorganic electrets. For ease of fabrication and compatibility with different substrate materials, polymer electrets with low process temperature have advantage over inorganic electrets such as SiO_2_ [1].

Tada [5] demonstrated a multipole rotational electret generator using Teflon-FEP having stable homocharges. He improved Jefimenko’s design [6], in which polarized poly-methyl methacrylate (PMMA) electret has unstable heterocharges. Boland et al. [7] adopted micro-electro-mechanical-system (MEMS) technology to the rotational power generator. Amorphous-fluorinated polymer Teflon AF was selected as the electret material, since it is compatible with the MEMS fabrication process. They obtained a surface charge density of -0.5 mC/m^2^ for a 9 μm-thick film. Lo and Tai [8] employed parylene HT as electret, and obtained the surface charge density as high as 3.69 mC/m^2^ for a 7.3 μm-thick film. Tsutsumino et al. [9] firstly proposed amorphous perfluoro polymer CYTOP (cyclic transparent optical polymer) as electret, and found that CYTOP with amidosyl end-group (CTL-M) can provide up to 1.37 mC/m^2^ for a 15 μm-thick film, which is 3 times larger than that of Teflon AF. Suzuki et al. [10] developed a MEMS-based vibration energy harvester with CTL-M and obtained 1 μW at 63 Hz. In our previous studies [11,12], we further improved the charging characteristic of CYTOP by adding excess aminosilane to form additive-based nano clusters in polymer (CYTOP-EGG). We have obtained the surface charge density of 2.0 mC/m^2^ for a 15 μm-thick film with high thermal stability of the implanted charges. Recently, Miyoshi et al. [13] developed a rotational electret energy harvester with CYTOP-EGG, and obtained up to 80 μW from arm swing during human walking.

Electret materials has been discovered by experimental trials, while their properties as electrets are not predicted. In order to develop a new high-performance electret, it is necessary to understand electron trap site and trap energy. The band gap model provides an intuitive image of charge trap in polymer system; electron/hole traps can exist between the conduction/valence band, and their distribution overlaps and forms density of state [1,14,15]. With this idea, one way to explore the charge trap in polymer is focusing on the morphology of the system. Various morphological structures can exist in bulk polymer system (e.g. crystalline, lamella, amorphous, etc.), and the mobility of trapped charge is known to depend on the local structure [16,17]. Research on polyethylene (PE) used as electric insulation provides detailed image for the phenomenon. Serra et al. [18] and Meunier and Quirke [19] have shown that electron affinity (EA) of PE is negative. This implies that a single PE molecule is naturally electrophobic, and extra electrons in PE system will easily move to low-volumetric-density region to be stabilized. Therefore, electron trap of PE strongly depends on its morphology. Wang et al. [20] studied single-electron traps in various structure motifs of PE by molecular dynamic simulation, and calculated their trap-related electronic properties with a block Lanczos algorithm. Their analysis shows that the more disorder is introduced to the structure (crystalline to amorphous), the more electrophobic is the system. With this idea in hand, one can qualitatively estimate property of bulk electret material based on configuration of the material.

Another useful approach is focusing on the intramolecular structure of a single molecule. Polymer molecules are twisted, entangled, and often react during manufacturing process. The formed local defects or impurities can become charge trap site [17]. Meunier et al. [21] and Huzayyin et al. [22] performed quantum mechanical analysis to investigate electron trap in PE. They utilized density functional theory (DFT) to analyze electronic state of PE molecules. By comparing analyzed EA of possible isomers and defects in the PE insulator, they found that traps caused by chemical impurities can be deeper than those caused by morphological disorder. Takada et al. [23] examined other polymers such as ethylene-tetra-fluoro-ethylene (ETFE), poly-tetra-fluoro-ethylene (PTFE), and polyimide. Their DFT analysis showed that local band structure of polymer chain can be used to distinguish intramolecular charge trap site.

In the present study, we focus on electron trapping behavior in amorphous fluorinated polymer. In our previous studies on CYTOP electrets [11,12], we found that electron-trapping characteristic of CYTOP strongly depends on their end-group, even the number of repeat unit is 270–540. Therefore, we investigate the effect of the end group on the electron trap by using a series of experiments and quantum mechanical analysis.

## 2. Experimental approach on electron trapping of CYTOP

**Figure 1.**
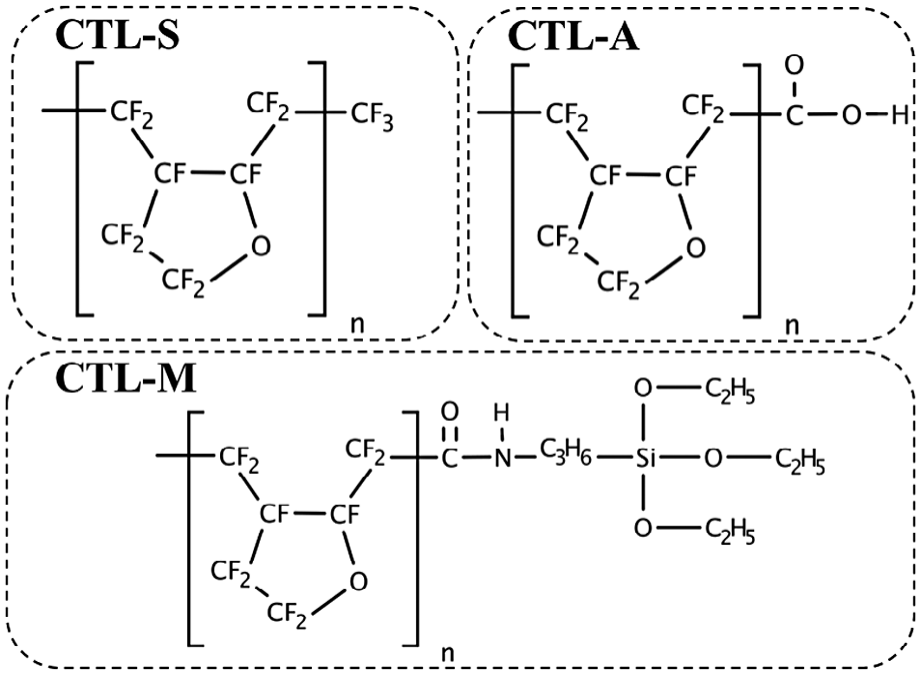
Schematic chemical structure of CYTOP polymers.

**Figure 2.**
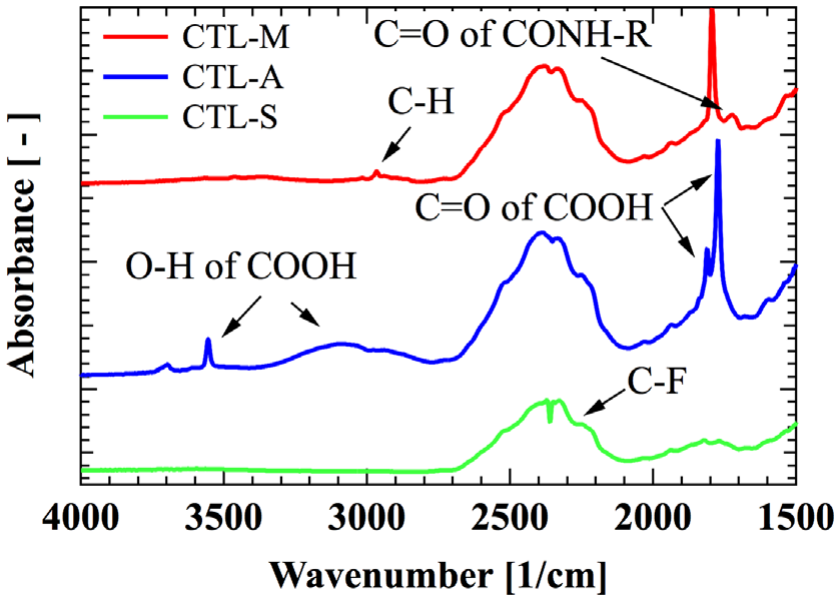
Infrared absorption spectra of CTL-S, CTL-A and CTL-M.

A high-performance polymer electret CYTOP is selected as the main target of our analysis. CYTOP is amorphous perfluoro polymer with pentagonal ring structure, which has favorable polymer electret characteristics such as high dielectric strength of 110 kV/mm and low dielectric coefficient of 2.1. Three types of CYTOP with different end-group are commercially available as shown in Figure 1; CTL-S has trifluoromethyl end, CTL-A has carboxyl end, and CTL-M has amidosyl end. Figure 2 shows infrared absorbance spectra of the CYTOP solid films, showing peaks for representative bonds. C-F overtone spectra from repeat units shows broad peak around 2400 cm^−1^ for every samples. Carboxyl group of CTL-A can be found from O–H stretch peaks at 3555 cm^−1^, 3100 cm^−1^ and C=O stretch peaks at 1773 cm^−1^, 1811 cm^−1^. In CTL-M, C=O stretch peak at 1725 cm^−1^ is resulted from amide bond, and a weak C–H peak at 2400 cm^−1^ originates from amidosyl branches.

**Figure 3.**
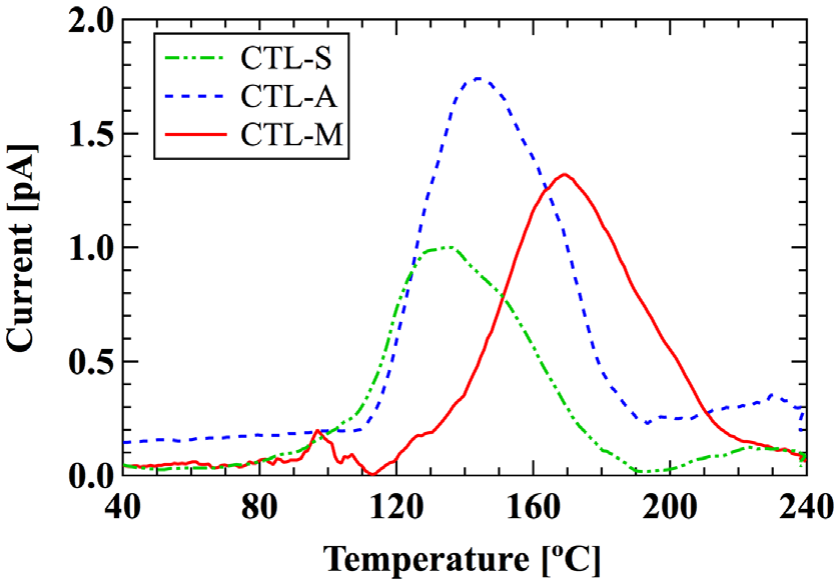
TSD spectra of CYTOP polymers.

Figure 3 shows the open-circuit thermally stimulated discharge (TSD) spectra of 15 μm-thick samples [11]. CYTOP films are spun on a 0.3 mm-thick copper plate with area of 30 × 30 mm^2^. The samples are charged with corona discharge. Applied voltages for the needle and the grid are −8 kV and −600 V, respectively. The area of TSD electrode is the same as that of the copper plate. The ramping rate is 1 °C/min. The peak temperature of CTL-S, CTL-A, and CTL-M are 135, 145 and 170 °C, showing that the order of thermal stability of trapped charges is CTL-M > CTL-A > CTL-S. Table 1 summarizes the surface charge density at 200 h after charging [11]. It is also shown that the surface potential is constant over 4000 h (not shown here) [11]. These results signify the long-term stability of trapped charge, which follows the order of TSD peaks; the electrons trapped in CTL-M is the most stable, while that of CTL-S is the least stable.

**Table 1.** Surface charge density of negatively charged, 15 μm-thick amorphous fluropolymers [11,12].

	End group	Surface charge density after 200 h (mC/m^2^)
CTL-M	Amidosyl	−1.3
CTL-A	COOH	−0.87
PTFE	–	−0.84
CTL-S	CF3	−0.37

**Figure 4.**
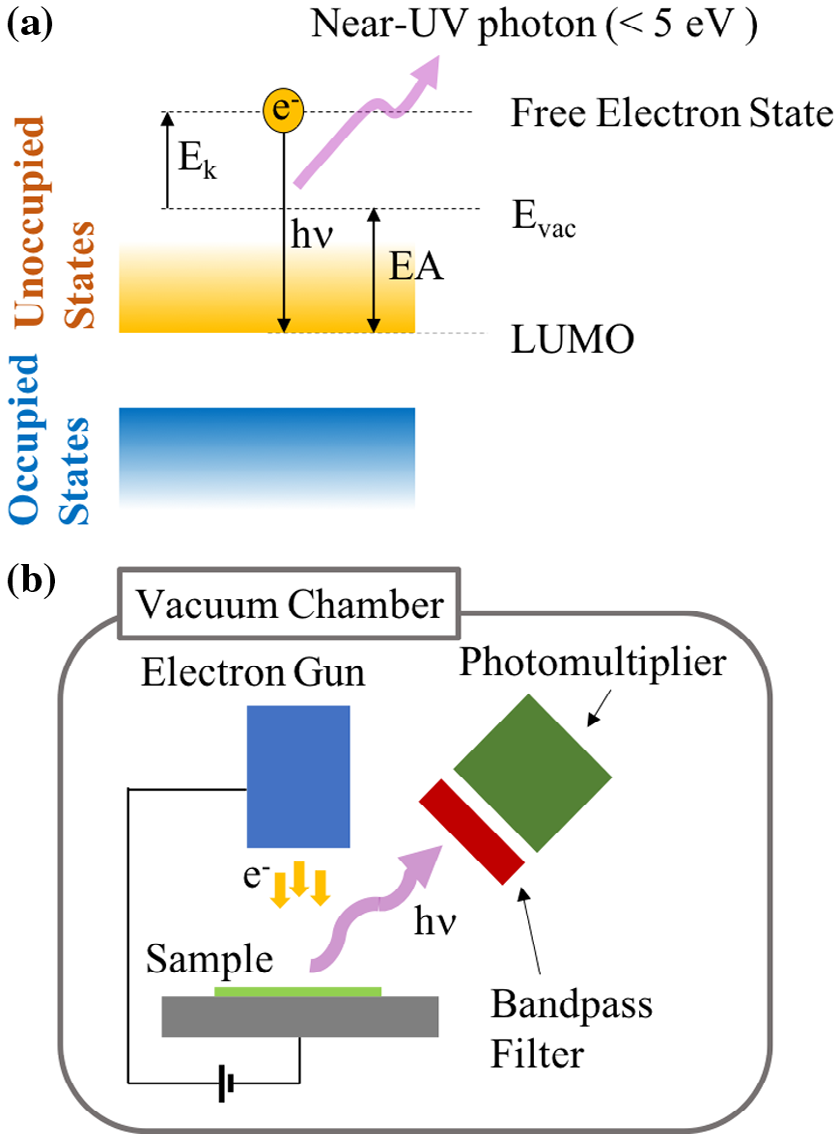
(a) Principle of the inverse photoelectron spectroscopy (LEIPS), (b) Schematic of the LEIPS measurement setup.

For investigating EA of electret material, we employ the low-energy inverse photoelectron spectroscopy (LEIPS) [24,25]. This technique examines the lowest unoccupied molecular orbital (LUMO) based on the inverse photoelectron spectroscopy but minimizes damage to organic samples by lowering the incident electron energy less than 5 eV, which is a typical damage threshold of organic molecules. Figure 4 shows the measurement principle and a schematic of LEIPS [26]. Monoenergetic electrons are injected to the sample surface. The incident electron entering a high-lying free-electron state transits to a low-lying unoccupied state, while emitting a photon having an energy equivalent to the difference of the two states. In the present study, photons having wavelength of 260 nm (4.77 eV in energy) are detected using a bandpass filter and a photomultiplier. The LEIPS spectrum is obtained by measuring photon intensity as a function of electron energy *E*_*k*_. The bias voltage applied between the electron gun and the sample is changed to control *E*_*k*_. Since electrons having kinetic energy higher than the vacuum level enter the substrate, the vacuum level of the sample *E*_vac_ can be determined from the onset of the electron current measured at the sample. EA is the edge of unoccupied states with respect to *E*_vac_.

LEIPS is usually applied to organic semiconductor materials. One of the biggest obstacles for measuring dielectric materials is sample charging. Electrons injected to a dielectric sample induce charge-up of the sample, which results in the shift of measured energy levels. The magnitude of energy shift depends on the number of electrons trapped in the sample. In the present study, we assume the energy shift is proportional to the thickness of dielectric layer. We measure the onset of LEIPS spectrum *E*_onset_ for samples with different film thicknesses. The unwanted effect of charge-up is compensated by extrapolating the measured data. We also perform measurements at different electron currents of 0.15 and 0.70 μA. The mean data are obtained from three separate measurements. It is noted that, although the tunneling conduction exists near the interface between bottom electrode and CYTOP, we expect that the number of trapped charges aside from the interface is linearly increased with the sample thickness, and mainly influence the energy shifts in our experiment.

The sample preparation process starts from arranging 10 mm × 10 mm p-doped silicon wafer with the thickness of 0.4 mm, treated with hydrofluoric acid in order to remove native oxide. Then, diluted CYTOP CTL-S solutions of different concentration are spun on at 4000 rpm. Finally, the samples are baked at 100 °C for 1 h followed by another bake at 200 °C for 1 h. Thicknesses of the CTL-S films measured with a surface profiler (Alpha-Step AS500, KLA Tencor) are 2.1, 4.1, 8.2 nm for concentration of 0.05, 0.1, and 0.2 %, respectively. Their standard deviation is within 0.1 nm.

**Figure 5.**
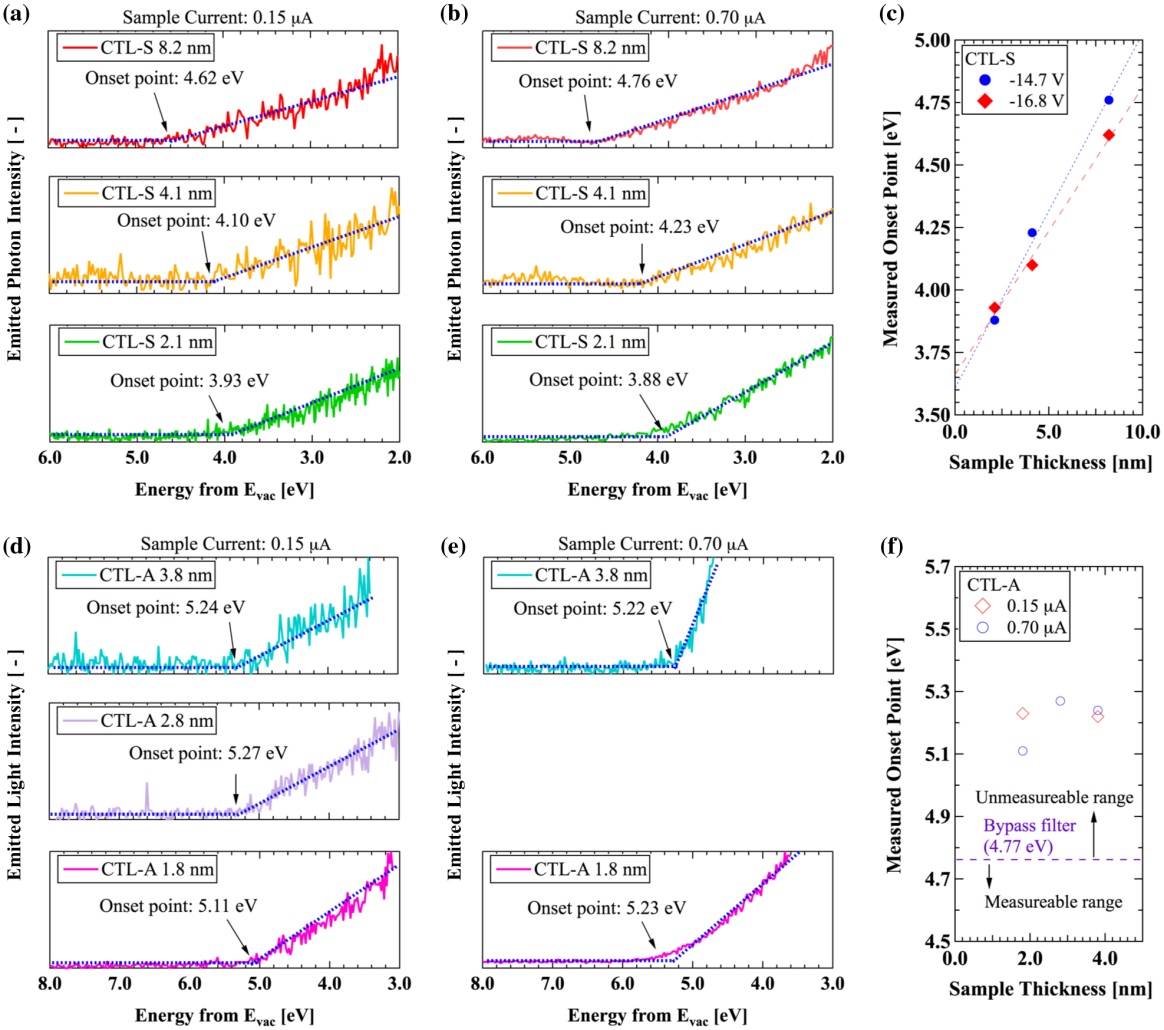
LEIPS results: (a) Intensity of emitted light from CTL-S with 0.15 μA of sample current, (b) Intensity of emitted light from CTL-S with 0.70 μA of sample current, (c) Thickness dependence of LEIPS onset energy for CTL-S, (d) Intensity of emitted light from CTL-A with 0.15 μA of sample current, (e) Intensity of emitted light from CTL-A with 0.70 μA of sample current, (f) Thickness dependence of LEIPS onset energy for CTL-A.

Figure 5(a) and (b) display the LEIPS spectra of CTL-S. The onset energy is considered as the representative EA including the charge-up effect. As explained above, we estimate the charge-up-free EA by extrapolating the measured data to zero film thickness. EA thus estimated is 3.608 eV at 0.70 μA sample current and 3.663 eV at 0.15 μA as shown in Figure 5(c). Because two separate measurement data with different sample current are in good agreement within the systematic error range of 0.2 eV, we can conclude that EA of CTL-S is 3.6 eV.

Figure 5(d)–(f) show the measurement data for the CTL-A samples having thickness of 1.9, 2.8, and 3.8 nm. It is found that the onset energies exceed the upper limit of present measurement system of 4.77 eV. The observed onset energy higher than the limit should be originated from unoccupied orbital other than LUMO. This fact indicates that EA of CTL-A should be higher than that of CTL-S. It is also found that the measurement data for CTL-M exhibit the same tendency as for CTL-A, showing that EA of CTL-M is also higher than that of CTL-S (not shown). In order to measure higher EA, a bandpass filter with higher threshold energy should be used, but the sensitivity becomes lower. The UV-induced discharge current measurement [27,28] might be useful, in which UV light is irradiated into a charged electret sample while sweeping the energy of photon.

## 3. Quantum mechanical approach on electron trapping

EA of electret is analyzed by calculating the energy of orbital where the electron is trapped. Conventionally-used bulk polymer electret (PE, ETFE, and PTFE) and CYTOP polymers are analyzed using DFT. In the present DFT calculation, Kohn-Sham equation shown in Equation (1) is iteratively solved by self-consistent method [29]. In the left-hand side, the first term expresses electron’s kinetic energy, the second considers Coulomb interaction from nucleus, the third is averaged Coulomb repulsion from other electrons. *V*_*XC*_ the exchange functional describing quantum mechanical interaction between electrons. Acquired *ε*_*i*_ is energy of *i*th orbital *ψ*_*i*_.

(1) $$\left(-\frac{1}{2}\nabla^{2}-\frac{Z_{A}}{r_{A}}+\int \frac{\rho \left(r^{\prime }\right)}{\left|r_{i}-r^{\prime }\right|}dr^{\prime }+V_{\mathrm{XC}}\right)\psi_{i}\left(r\right)=\varepsilon_{i}\psi_{i}\left(r\right)$$

In Koopmans’ theorem, if the extra electron is trapped in *ψ*_*i*_, the opposite sign of *ɛ*_*i*_ can be considered as a vertical EA, which is EA disregarding change of geometry before/after electron addition [30,31]. Long-range corrected DFT (LC-DFT) complements *V*_*XC*_ with additional term considering electron-exchange interaction from distant electrons and enables quantitative evaluation of the orbital energy [31,32]. Among various LC-DFT methods, we adopted LC-BLYP functional in the present study, since LC-BLYP is able to describe the polarizability and the orbital energy well, especially for fluorine-rich polymers including CYTOP [33–35].

Isomer analysis is firstly performed in order to find representative structure. We prepared possible monomer and dimer structures of targets: PE, ETFE, PTFE, CTL-S, CTL-A, and CTL-M. Preliminary geometries are prepared in molecular dynamic simulation with universal force field parameters [36], finding the most stable structure at 0 K condition. Quantum mechanical (QM) geometry optimization is followed with NWChem [37] with 6-31+g* basis set to discover an initial ground-state structure. After that, we changed dihedral angles of interest from 0° to 360° by every 45°. QM geometry optimization is followed to changed structures, and the energy of ground-state molecular system after optimization is compared. The structure having the most stable energy among them is used for further study. With the representative structures in hand, we build chain structures made from 8 carbons for PE, ETFE, and PTFE. Monomer and tetramer structures are prepared for CTL-S, CTL-A and CTL-M. Then, an extra electron is added to the systems and geometry optimization is performed once again to find stable ground-state structure of negatively-charged state (−1).

**Figure 6.**
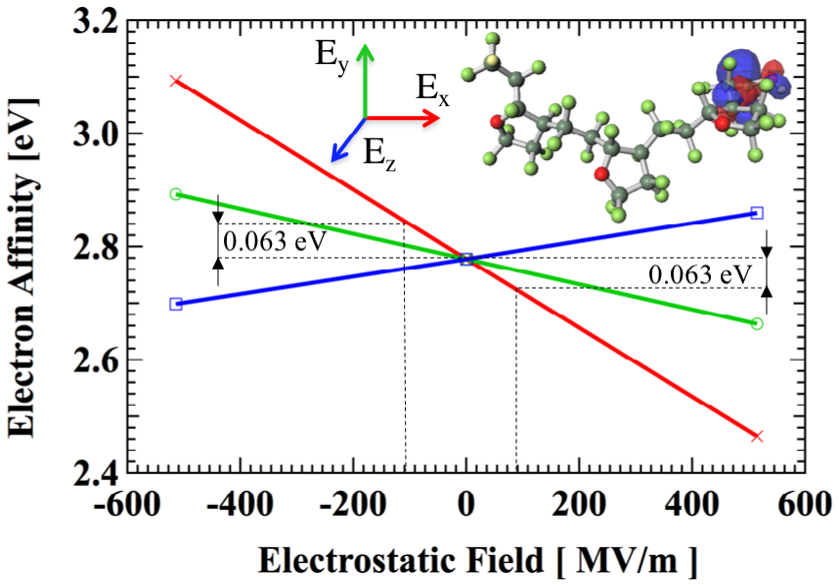
Influence of the external electrostatic field on electron affinity.

When the electret is charged, strong electrical fields up to 100 MV/m are imposed. A preliminary simulation with GAMESS [38] using unrestricted Hartree-Fock method and 6-311G** basis sets is performed to investigate the effect of electrical field on EA. A CTL-A trimer anion is firstly prepared with geometry optimization. Then, we apply external electrostatic field in three orthogonal directions. EA is calculated from the energy of orbital where the electron is trapped. As shown in Figure 6, EA without external electrostatic field and with ±514.2 MV/m are acquired. When the electrostatic field of ±100.0 MV/m is applied, the change of EA is within 0.063 eV, which is only 2.3% of EA of 2.78 eV in a neutral field. Therefore, in the following simulation, we can safely neglect the effect of external electrostatic field.

**Figure 7.**
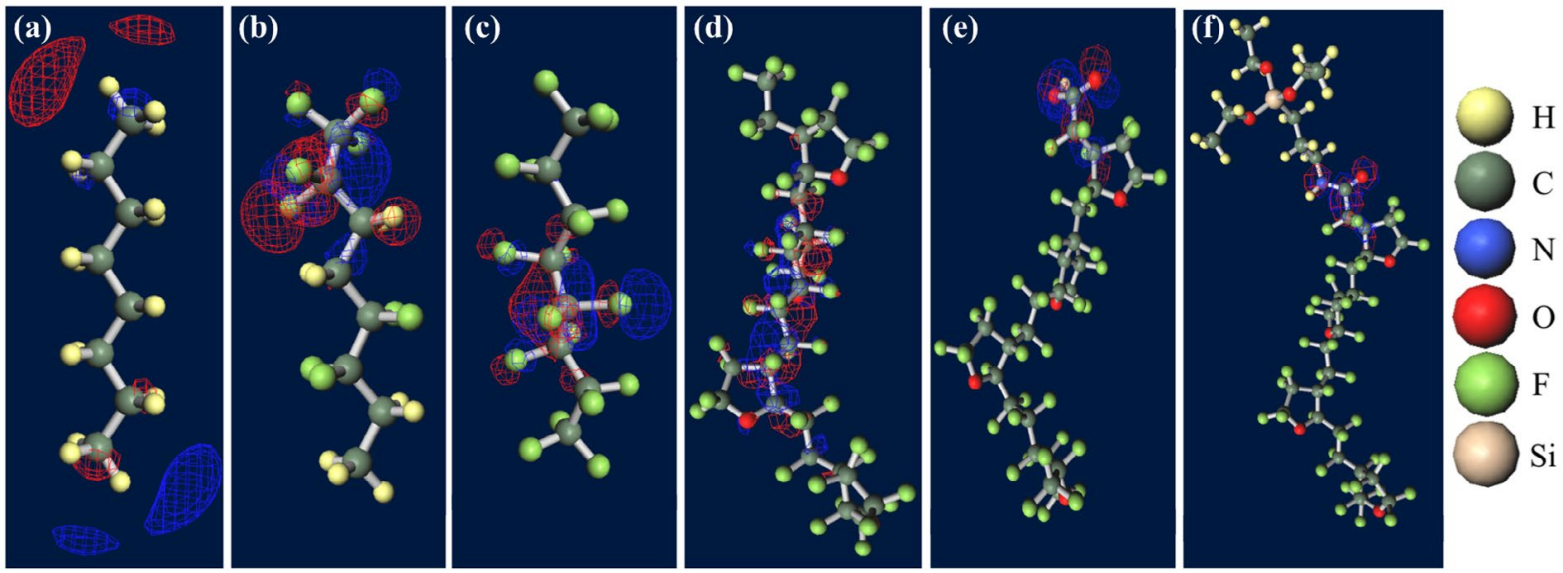
Ground-state structures of (a) PE, (b) PTFE, (c) PTFE, (d) CTL-S, (e) CTL-A, (f) CTL-M.

Note: Red and blue meshes represents distribution of the trapped electron.

**Figure 8.**
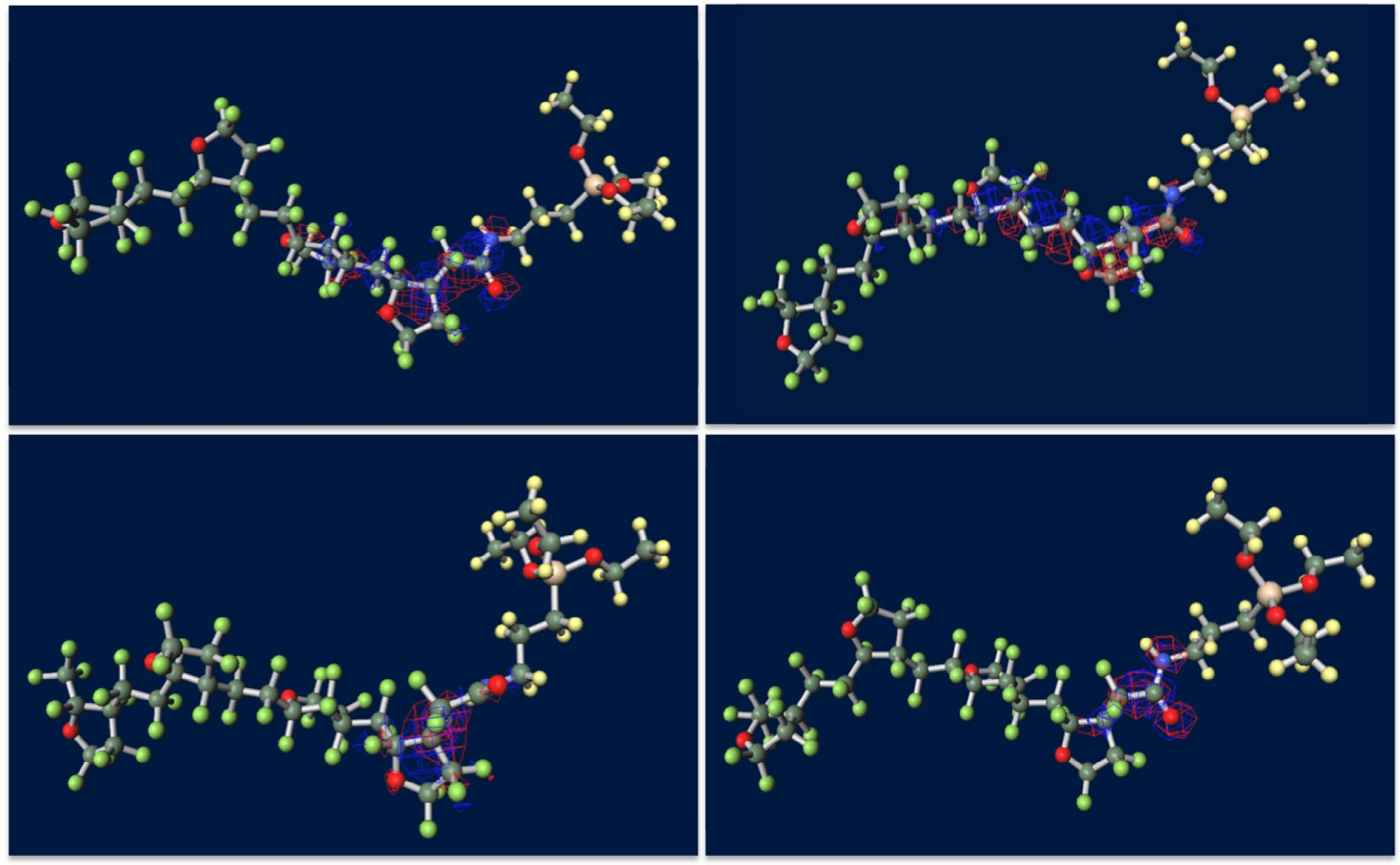
Strong electron-attracting characteristics of CTL-M end group.

Note: The trapped electron stays near amide bond of CTL-M, even structural conformation is transformed.

Figure 7 shows ground-state structures and distribution of the trapped electron visualized by Winmostar (X-ability). Electron trapped in PE does not lie on structure and exists in exterior of the chain, implying that this chain-like PE is electrophobic. Clear tendency of electron concentration near fluorine-rich region is shown in ETFE and PTFE. From the results of CTL-A and CTL-M, distinguishing inclining of trapped electron near end-group is found. In CTL-S, the trapped electron broadly populates along the structure. Meanwhile, it is lying on the carboxylic end in case of CTL-A and concentrated to the amino bond for CTL-M. Additionally, we have prepared four randomly deformed structures of CTL-S, CTL-A and CTL-M tetramers to find change of electron distribution after transformation. Figure 8 shows trapped electron distribution at CTL-M tetramers having different conformations. The simulation result shows strong electron attraction of amidosyl end-group in CTL-M again. The trapped electron in CTL-M is kept around the amide bond, although the trapped charge distribution is somewhat diffused. On the other hand, trapped electron in CTL-S often jumps to neighboring repeat units when the conformation is changed.

**Figure 9.**
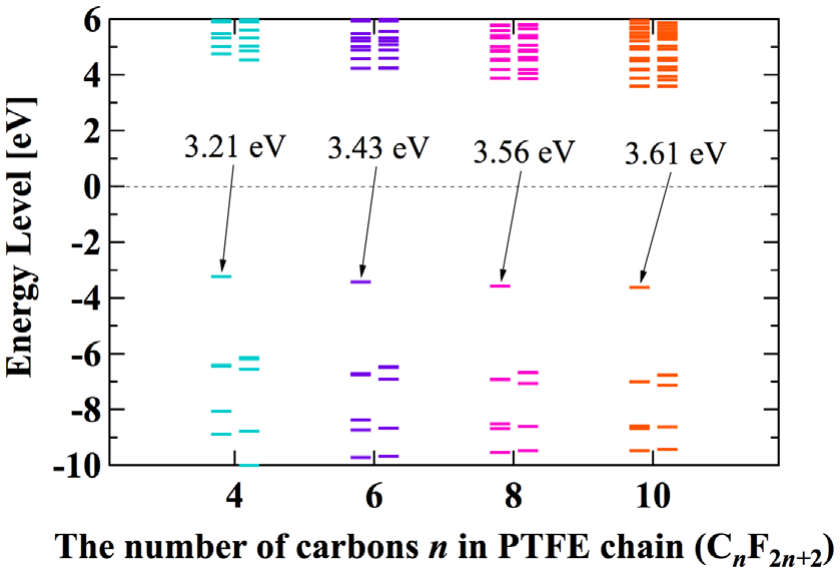
Calculated orbital energy level of PTFE with different number of carbons.

For analysis of EA of PE, ETFE, and PTFE, we use the chain structures with 8 carbons. This is because these structures are already in the global minimum state, and EA of chain-like PE seems to saturate after the number of carbon is above 8 [39]. Figure 9 shows the electron for different number of carbons from 4 to 10. The bars in Figure 9 represent the calculated orbital energy. The bars with an arrow correspond to the energy of orbital where the additional electron is trapped and its opposite sign corresponds to the vertical EA. The bars below are occupied orbitals, while upper bars are unoccupied orbitals. The zero level can be interpreted as the boundary index. Trapped electron having negative energy level is expected to be stable, while having positive level is unstable and easily escape from the molecule. The EA of PTFE with 8 carbons (3.56 eV) is close to that of 6 carbons (3.43 eV) and 10 carbons (3.61 eV).

**Figure 10.**
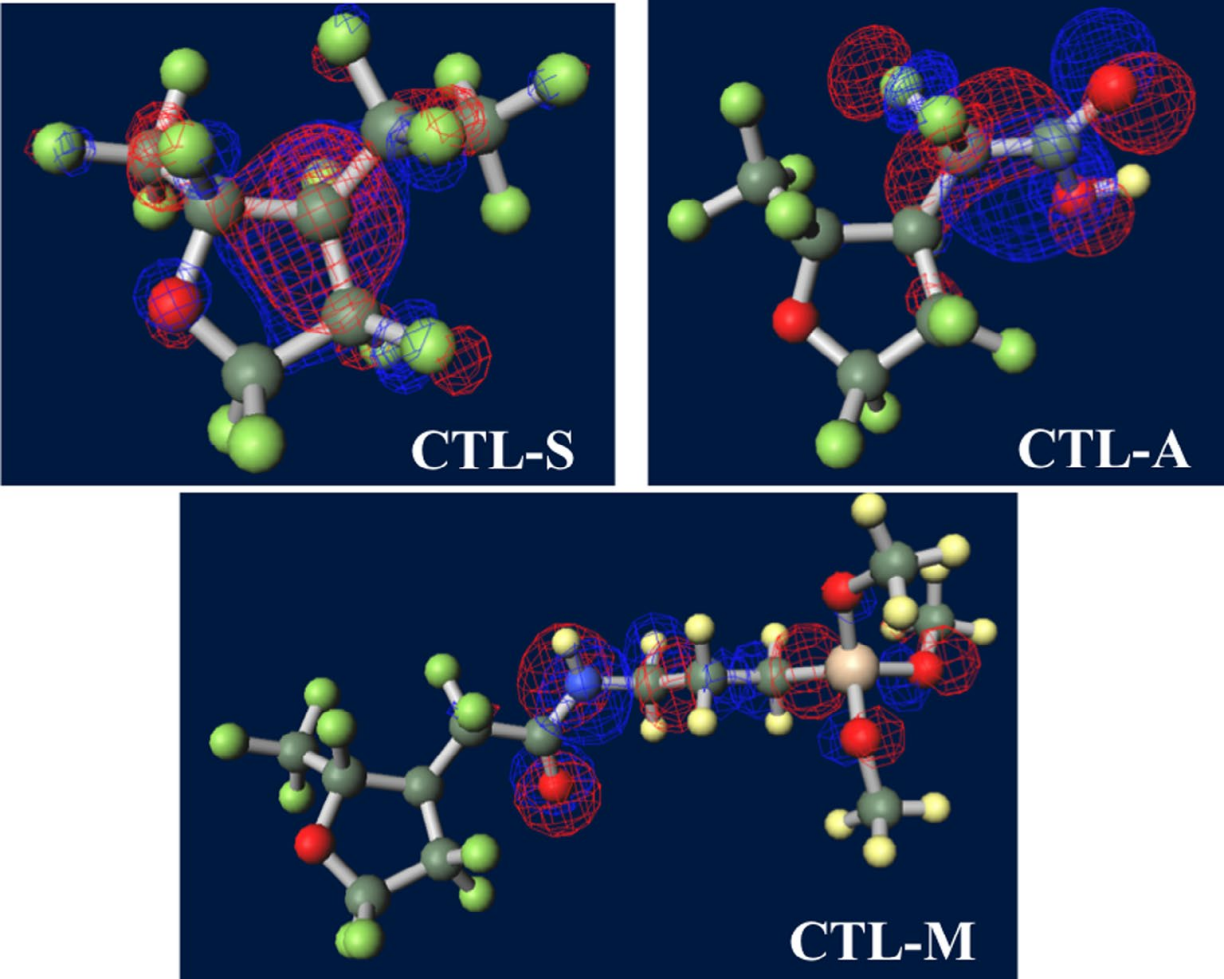
Visualized distribution of the trapped electron in CYTOP monomers.

**Figure 11.**
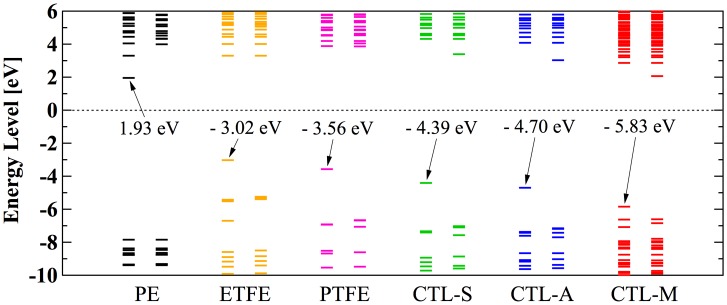
Calculated orbital energy level of polymer electrets.

Note: The bars with arrows represent energy of orbital where the electron is trapped.

However, in our CYTOP tetramer systems, we found that it is difficult to find the global minimum state, because of abundant degrees of freedom in configuration. Our calculation often falls into the local minimum and unable to reach the global minimum state, although we use a quasi-newton geometry optimization algorithm to find the most stable structure based on the initial structure. Therefore, we use monomer units instead of tetramer units for CTL-S, CTL-A, and CTL-M. It is expected that EA of CYTOP is relatively insensitive to the number of units, because the trapped electron is located near the end group. Note also that, for poly-para-phenylene-vinylene, EA for different number of repeat units is within 0.5 eV [40]. Figure 10 displays negatively-charged monomer systems of CTL-S, CTL-A, and CTL-M with the distribution of its trapped electron. The distributions of electron have similarities with the result shown in Figure 7; electron in CTL-A and CTL-M is biased towards to the end-group side. The orbital energies of polymers examined in this study are presented in Figure 11. Calculated EA shows clear order as follows: CTL-M (5.83 eV) > CTL-A (4.7 eV) > CTL-S (4.39 eV) > PTFE (3.56 eV) > ETFE (3.02 eV) > PE (−1.93 eV). Although the order of EA for PE, ETFE and PTFE in the present analysis is in accordance with that of the TSD peaks, we can obtain only qualitative information based on the present analysis. This is because those polymers consist of heterogeneous local morphology populations (e.g. crystalline, amorphous, lamella, etc.), and their electret properties strongly depend on the system morphology [16,17,20].

For CYTOP polymers, the order of calculated EA corresponds well to that of TSD peak in Table 2 (CTL-M > CTL-A > CTL-S). Although our analysis on single-molecular charge trap site cannot perfectly interpret the real system of electret, we expect our method can be used as an indicator to predict performance of amorphous polymer electrets.

**Table 2.** TSD peak temperature of polymer electrets.

	PE	ETFE	PTFE	CTL-S	CTL-A	CTL-M
	[42,43]	[44]	[12,43,45]	[11,12]	[11,12]	[11]
Sample thickness (μm)	1, 10, 160	50	15, 25	15	15	15
TSD peak temperature (°C)	35–70	120–160	138–230	135	148	170

It is noted that calculated EA of CTL-S is 4.39 eV, which is 0.8 eV higher than the measured value of 3.6 eV shown in Figure 5. This difference is mainly originated from the geometries used for the present analysis. In real polymer systems, the charge trap sites have various conformations different from our ground state structure. The effect of the other molecules surrounding the trap site by their electrostatic multipole interaction [41] will also affect the discrepancy.

## 4. Conclusions

In the present study, electron affinity (EA) of CYTOP polymers with different end group is examined through a series of experiments and DFT simulations. EA of CTL-S has been measured by low-energy inverse photoelectron spectroscopy (LEIPS). After careful examination of the dependences of LEIPS spectra on the film thickness and sample current, EA of CTL-S is determined to be 3.6 eV. It is also found that EA of CTL-A and CTL-M is higher than that of CTL-S. Quantum mechanical analysis of trapped electron in polymer electrets is also performed. Amidosyl end-group in CYTOP CTL-M is found to act as a powerful charge trap, even more than perfluorinated main structure itself. The order of EA obtained with the present simulation is CTL-S < CTL-A < CTL-M, which is in qualitative agreement with the TSD peak temperature, showing applicability of the present methods for predicting charge trapping characteristics of amorphous polymer electrets toward higher performance.

## Disclosure statement

No potential conflict of interest was reported by the authors.

## Funding

This work was partially supported by JST CREST [grant number JPMJCR15Q3], Japan. SK is supported through the Leading Graduates School Program, ‘Global Leader Program for Social Design and Management’ by MEXT, Japan.
